# Genetic polymorphisms of angiotensin-2 type 1 receptor and angiotensinogen and risk of renal dysfunction and coronary heart disease in type 2 diabetes mellitus

**DOI:** 10.1186/1471-2369-10-9

**Published:** 2009-03-27

**Authors:** Julie Lin, Frank B Hu, Lu Qi, Gary C Curhan

**Affiliations:** 1 Channing Laboratory, Department of Medicine, Brigham and Women's Hospital, Harvard Medical School, Boston, MA, USA; 2 Renal Division, Department of Medicine, Brigham and Women's Hospital, Harvard Medical School, Boston, MA, USA; 3 Department of Nutrition, Harvard School of Public Health, Boston, MA, USA; 4 Department of Epidemiology, Harvard School of Public Health, Boston, MA, USA

## Abstract

**Background:**

Increased activation of the renin-angiotensin system (RAS) may be important in promoting coronary heart disease (CHD) and renal dysfunction, but limited data are available on associations between angiotensin type 1 receptor (*AGT1R*) and angiotensinogen (*AGT*) genotypes in type 2 diabetes.

**Methods:**

Study participants were diabetics from the Health Professionals Follow-Up Study (HPFS) and the Nurses' Health Study (NHS). We analyzed single nucleotide polymorphisms (SNPs) associated with cardiovascular pathophysiology (including *AGT1R *T573C, *AGT1R *A1166C, and *AGT *M235T) and presence of renal dysfunction (eGFR<60 ml/min/1.73 m^2^) or history of CHD.

**Results:**

The *AGT1R *1166 C-allele was associated with eGFR<60 ml/min/1.73 m^2 ^(multivariable OR 1.63 [1.01, 2.65]) in the HPFS men (n = 733) and in the combined dataset (n = 1566) (OR 1.42 [1.02, 1.98]). The *AGT1R *1166 C-allele was also associated with CHD in men (OR 1.57 [1.10, 2.24]). In NHS women (n = 833), *AGT *235T-allele was associated with CHD (OR 1.72 [1.20, 2.47]). Removal of hypertension from the fully adjusted models did not influence results, suggesting that the associations may not be mediated by hypertension. There were significant interactions between sex and *AGT1R *1166 C-allele (p = 0.008) and *AGT *M235T (p = 0.03) in models for CHD. No significant associations were seen between *AGT1R *T573 C-allele and renal dysfunction or CHD.

**Conclusion:**

Polymorphisms in *AGT1R *and *AGT *genes are associated with renal dysfunction and CHD in type 2 diabetes and further support the important role of the RAS in these complications. Sex may modify associations between *AGT1R *1166 C-allele and *AGT *235T and CHD in type 2 diabetes.

## Background

Increased activation of the renin-angiotensin system (RAS) has been postulated to play a central role in the progression of chronic kidney disease (CKD) and coronary heart disease (CHD). This theory is supported by randomized clinical trials that have shown beneficial clinical effects of the blockade of production of angiotensin-II (AII) by angiotensin converting enzyme inhibitor (ACE-I) medications or antagonism of AII action through angiotensin receptor blockade (ARBs). For example, among diabetics, the ARB losartan significantly slowed progression of CKD [1], whereas the ACE-I medication ramipril significantly reduced CHD morbidity and mortality [2].

Although the D-allele of the *ACE *I/D polymorphism has been associated with diabetic nephropathy in two large meta-analyses of several thousand individuals [3,4], the role of genetic polymorphisms in other components of the RAS in CKD and CHD in people with type 2 diabetes has been less well defined. Associations between these SNPs and renal and cardiovascular diseases have been reported in other sub-groups, however. For example, the homozygous *AGT *(angiotensinogen gene) 235-T/T genotype has been associated with faster progression to ESRD in patients with glomerulonephritis [5] and with susceptibility to nephropathy in patients with type I diabetes mellitus [6], although not in two populations with type 2 diabetes [7,8].

Higher concentrations of plasma angiotensinogen are associated with the *AGT *235 T-allele [9] and may be directly related to the number of Thr alleles present [10] thereby supporting the theory that components in the RAS are physiologically activiated and may promote glomerulosclerosis and interstitial fibrosis via accumulation of extracellular matrix [11]. Recent research has revealed that another more clinically relevant *AGT *polymorphism may be a linked promoter substitution that increases the rate of gene transcription; this G-6A polymorphism appears to be the one most strongly associated with hypertension [12]. Genetic variation in *AGT *genes, especially the *AGT *235T and AGT (-6) G alleles have also been reported to be associated with CHD [10,13,14].

Angiotensin II type 1 receptor (AT1R) polymorphisms may influence intrarenal angiotensin II (AngII) activity. Healthy Caucasians carrying the A1166 C-allele (AC or CC) polymorphism showed lower basal GFR and basal renal plasma flow and had increases in GFR following treatment with the AT1R blocker losartan [15].

We therefore investigated associations of genetic polymorphisms in the *AGT1R *and *AGT *genes including *AGT1R *T573C, *AGT1R *A1166C, and *AGT *M235T and presence of CKD and CHD in type 2 diabetes.

## Methods

### Participants

#### Health Professionals' Follow-Up Study

The Health Professionals' Follow-Up Study (HPFS) was established in 1986 when 51,529 U.S. male health professionals, aged 40 to 75 at study initiation, returned a mailed questionnaire providing information about diet, lifestyle factors, and medical history [16]. Participants were mailed follow-up questionnaires every two years to update information. In 1993–1994, 18,159 participants provided blood samples that were shipped on ice by overnight delivery and stored at -130 degrees Celsius as previously described [17]. Diabetes mellitus (DM) was first identified by self-report on a biennial questionnaire and confirmed by a Diabetes Supplemental Questionnaire (DSQ) in 2000; the validity of the DSQ in confirming DM has been demonstrated in the HPFS cohort [18]. The HPFS DM blood cohort consists of 1000 men with confirmed diabetes at baseline who provided a blood sample in 1993–1994. Exclusion criteria for the current analyses were as follows: a) age of onset of DM ≤ 25 years of age (to attempt to restrict the study to type 2 DM) (n = 31), b) subjects with a reported date of DM diagnosis after the date of blood draw (n = 224), c) participants who reported on the DSQ that they were on dialysis (n = 9) or had a kidney transplant (n = 1), d) serum creatinine > 5.0 mg/dl (n = 1), and e) serum creatinine ≤ 0.5 mg/dl (felt to be physiologically implausible) (n = 1). After these exclusions, 733 men were available for analysis.

#### Nurses' Health Study

The NHS was initiated in 1976 with the enrollment of 121,700 U.S. nurses aged 30–55 years. This cohort is followed through questionnaires related to lifestyle factors and health outcomes that are mailed biennially. In 1989, 32,826 participants provided blood samples that were shipped on ice by overnight delivery and stored at -130 degrees Celsius as previously described [19]. Of these study participants who provided blood samples in 1989–1990, 1,198 who had a confirmed diagnosis of type 2 diabetes were chosen as a NHS diabetes blood sub-cohort. Exclusion criteria for the current analyses were: a) age of onset of DM ≤ 25 years of age (to attempt to restrict the study to type 2 DM) (n = 39), b) subjects with a reported date of DM diagnosis after the date of blood draw (n = 304), and c) serum creatinine ≤ 0.5 mg/dl (likely to be a laboratory error and physiologically implausible) (n = 22). No NHS participants reported on the DSQ being on dialysis or having had a kidney transplant. After these exclusions, 833 women had data available for analysis.

This study was approved by the Partners' Healthcare Brigham and Women's Hospital Human Research Committee Institutional Review Board.

### Assessment of covariates

Race was initially reported on the 1986 questionnaire for HPFS and in 1992 for NHS. Other clinical and lifestyle variables were derived from data from the questionnaire closest to the blood collection, i.e. the 1994 questionnaire for HPFS and the 1990 questionnaire for NHS. Body mass index (BMI) was calculated by weight (kg/m^2^). A weekly metabolic-equivalent (MET) score was calculated from the physical activity questions. Self-reported hypertension [18] and smoking status [20] have also been previously validated in these cohorts.

### Laboratory Methods

#### Single-nucleotide polymorphisms (SNP) analyses

DNA was extracted from the buffy coat fraction of centrifuged blood using the QIAmp Blood Kit (Qiagen, Chatsworth, CA). All SNPs were genotyped using Taqman SNP allelic discrimination by means of an ABI 7900 HT (Applied Biosystems, Foster City, CA). Undetermined SNP results, which comprised of <10% of data for any individual SNP, were treated as missing values. The candidate SNPs investigated here were *AGT1R *T573C (rs5182), *AGT1R *A1166C (rs5186), *AGT *M235T (rs699), and *AGT *A(-6)G (rs5051). A concordance rate of 100% was seen in 108 blinded quality control samples for all SNPs assayed.

#### Estimation of kidney function

Plasma creatinine was measured by a modified kinetic Jaffe reaction with a coefficient of variation of 22% for HPFS and 10% for NHS. Renal function was estimated by the simplified MDRD equation where estimated glomerular filtration rate (eGFR) (ml/min/1.73 m^2^) = 186 × [PCr (mg/dl)]^-1.154 ^× [Age]^-0.203 ^× [0.472 if female] × [1.21 if black] [21] and the Cockcroft-Gault equation where estimated creatinine clearance (CrCl) = ([140-age (years) × weight (kg) × (0.8 if female)]/(Pcr × 72)) [22]. Moderate renal dysfunction was considered to be eGFR < 60 ml/min/1.73 m^2 ^or CrCl < 60 ml/min. Because results using CrCl < 60 ml/min were very similar to those using eGFR < 60 ml/min/1.73 m^2^, we present only the eGFR analyses.

### Ascertainment of Coronary Heart Disease

Coronary heart disease was defined as history of confirmed myocardial infarction, or self-reported coronary revascularization or angina. These self-reported outcomes have been previously validated by medical chart review [23].

### Statistical Analyses

In our primary analyses, we tested for associations of SNPs and outcomes using a dominant model where the presence of the disease allele (listed second in the SNP name as per convention) was considered the exposure of interest. We also examined recessive models, where the homozygous disease genotype was compared to all others, as well as additive genetic models, where the impact of each additional presumed disease allele was assessed.

Unconditional logistic regression was used to calculate odds ratios for GFR < 60 ml/min/1.73 m^2 ^or CrCl < 60 ml/min or presence of CHD. Multivariable models were adjusted for age (continuous, years), hypertension (yes/no), BMI (continuous), cigarette smoking status (never, past, current), physical activity (quartiles, METS/week), duration of type 2 diabetes (quartiles, years), and measured HgbA1c (quartiles). In the analyses of the combined cohorts, sex was included as a covariate in addition to all those variables included in fully-adjusted models stratified by sex. Coronary heart disease (yes/no) was also included as a covariate in models where eGFR < 60 ml/min/1.73 m^2 ^or CrCl < 60 ml/min were the outcome of interest. Estimated GFR < 60 ml/min/1.73 m^2 ^was included as a covariate for CHD analyses. All analyses were performed with SAS software, version 9.1 (SAS Institute, Inc., Cary, North Carolina).

## Results

Demographic and clinical information for HPFS and NHS participants with Type 2 diabetes are shown in Table 1. Participants were predominantly Caucasian, the majority was overweight or obese, and the mean time since diagnosis of type 2 diabetes was 10 to 11 years. Most participants had well-preserved renal function: only 90 HPFS men and 80 NHS women had eGFR < 60 ml/min/1.73 m^2^. Approximately 25% of participants had prevalent CHD. Only 38 (5%) participants in HPFS and 42 (5%) participants in NHS had both eGFR<60 ml/min/1.73 m^2 ^and CHD.

**Table 1:** Demographic and clinical characteristics of type 2 diabetics in the Health Professionals Follow-Up Study (HPFS) and Nurses' Health Study (NHS)

	HPFS in 1994 (n = 733)	NHS in 1990 (n = 833)
**Age (years)**	65.5 ± 7.9 (47–80)	59.5 ± 6.3 (43–70)
**African-American**	11 (1.5)	12 (1.4)
		
**Hypertension**	399 (45.6)	568 (68.2)
**Weight (kg)**	88.1 ± 16.2 (56.8–210.9)	80.4 ± 18.1 (38.6–154.5)
**Body Mass Index (BMI) (kg/m**^2^**)**	27.8 ± 4.4 (18.3–56.5)	29.9 ± 6.2 (15.1–54.9)
**BMI categories (kg/m**^2^**)**		
**<22**	33 (4.5)	78 (9.3)
**22–24.9**	160 (21.8)	102 (12.2)
**25–27.9**	229 (31.2)	155 (18.6)
**28–29.9**	131 (17.9)	110 (13.2)
**≥ 30**	180 (24.6)	384 (46.1)
		
**Activity (METs/week)**	29.6 ± 33.0 (0–228.8)	15.3 ± 20.3 (0.2–190.7)
		
**Cigarette smoking**		
**Current**	43 (5.8)	113 (13.6)
**Past**	392 (53.5)	341 (40.9)
**Never**	261 (35.7)	377 (45.3)
**Missing**	37 (5.1)	2 (0.2)
		
**Time since diagnosis of Type 2 diabetes (years)**	11.2 ± 9.1 (0.1–41.1)	10.3 ± 8.0 (0.1–41.1)
**Measured HbA1c (%)**	7.5 ± 1.6 (4.8–15.6)	7.2 ± 1.8 (4.4–15.4)
		
**Baseline coronary heart disease (MI, revascularization, or angina)**	194 (26.5)	216 (25.9)
**ACE-inhibitor medication use**	60 (8.2)	81 (9.7)
**Statin medication use**	48 (6.6)	Not available
		
**Measured plasma creatinine (mg/dl)**	1.1 ± 0.2 (0.6–2.9)	0.8 ± 0.2 (0.5–2.4)
**Creatinine clearance (ml/min)**	91 ± 30 (22–241)	100 ± 33 (17–244)
**eGFR (ml/min/1.73 m**^2^**)**	79 ± 18 (23–142)	83 ± 19 (22–139)

Results expressed as mean ± SD (range 0 to 100%) or no. (%)

ACE = Angiotensin-converting enzyme

eGFR= Estimated glomerular filtration rate

*AGT *A(-6)G is in complete linkage disequilibrium with M235T and results were almost identical between the two SNPs; therefore, we present only the results for *AGT *SNPs from the M235T analyses. Prevalence of the disease-associated allele ranged from 47% for *AGT1R *1166 C-allele to 68% for the *AGT1R *573 C-allele. Prevalence of homozygosity for the disease-associated allele ranged from 9% for *AGT1R *1166 C-allele to 20% for the *AGT1R *573 C-allele. The SNPs were in Hardy-Weinberg equilibrium (all p-values for chi-square test ≥ 0.60).

**Figure 1:**
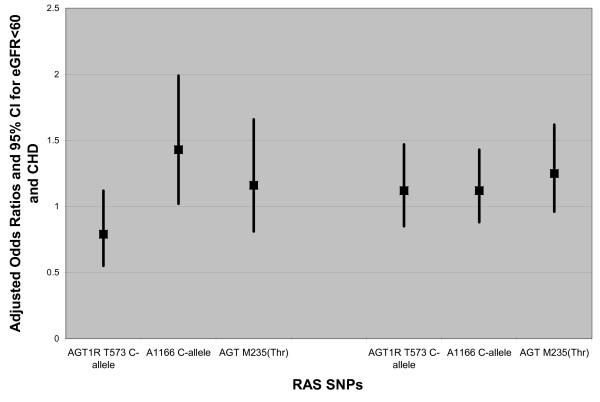
**Adjusted odds ratios for dominant model RAS SNPs and eGFR<60 ml/min/1.73 m^2 ^and CHD in combined cohort of HPFS (men) and NHS (women) with type 2 diabetes**. Error bars represent 95% confidence intervals.

For all SNPs, the magnitude of the fully adjusted odds ratios for the dominant models showed little difference when compared to the age-adjusted only models (Table 2). For the outcome of eGFR < 60 ml/min/1.73 m^2^, a significantly elevated odds ratio was seen in the fully-adjusted model for the *AGT1R *1166 C-allele in the HPFS men only (Table 2). Adjusted dominant models that did not include prevalent hypertension for the outcome of eGFR < 60 ml/min/1.73 m^2 ^were not different from the fully adjusted models. When the two cohorts were combined, the *AGT1R *1166 C-allele remained significantly associated with eGFR < 60 ml/min/1.73 m^2 ^(adjusted OR 1.42 [1.02, 1.98]) (Figure 1); no significant associations were seen in recessive or additive models in the combined dataset. Results using CrCl<60 ml/min as the outcome of interest were similar to those using eGFR < 60 ml/min/1.73 m^2 ^(results not shown).

**Table 2:** Logistic regression dominant models for association of RAS SNPs with presence of moderate renal dysfunction (GFR < 60 ml/min/1.73 m^2^) or CHD

	HPFS (n = 733)			NHS (n = 833)			
	**Number of cases/Total number with allele (%)**	**Age-adjusted OR** **[95% CI]**	Multivariable OR* [95% CI]	**Number of cases/Total number with allele (%)**	**Age-adjusted OR** **[95% CI]**	Multivariable OR* [95% CI]	p-value for sex *SNP interaction
***For GFR <60 ml/min/1.73 m^2^***							

**AGT1R T573C (C-allele)**	54/480 (11%)	0.68 [0.42, 1.09]	0.60 [0.36, 1.00]	68/569 (12%)	1.11 [0.68, 1.82]	0.96 [0.57, 1.61]	0.21
**AGT1R A1166C (C-allele)**	51/346 (15%)	1.59 [0.99, 2.49]	***1.63*** ***[1.01, 2.65]***	50/402 (12%)	1.18 [0.76, 1.83]	1.28 [0.80, 2.04]	0.54
**AGT M235T (Thr)**	57/469 (12%)	0.98 [0.61, 1.56]	0.91 [0.55, 1.49]	68/527 (13%)	1.62 [0.99, 2.69]	1.46 [0.85, 2.50]	0.25
***For prevalent CHD***							

**AGT1R T573C (C-allele)**	133/480 (28%)	1.26 [0.85, 1.85]	1.36 [0.91, 2.02]	148/569 (26%)	1.07 [0.75, 1.53]	1.02 [0.69, 1.49]	0.45
**AGT1R A1166C (C-allele)**	105/346 (30%)	***1.62*** ***[1.14, 2.29]***	***1.57*** ***[1.10, 2.24]***	99/402 (25%)	0.84 [0.61, 1.16]	0.85 [0.61, 1.20]	***0.008***
**AGT M235T (Thr)**	120/469 (26%)	0.95 [0.66, 1.35]	0.92 [0.64, 1.33]	154/527 (29%)	***1.72*** ***[1.20, 2.47]***	***1.66*** ***[1.13, 2.43]***	***0.03***

*Multivariable models are adjusted for age (continuous, years), hypertension (yes/no), BMI (continuous), cigarette smoking status (never, past, current), physical activity (quartiles, mets/week), time since Type 2 diabetes diagnosis (quartiles, years), and measured HbA1c (quartiles). Multivariable models for renal dysfunction are adjusted for presence of coronary heart disease (yes/no) and multivariable models for CHD are also adjusted for eGFR<60 ml/min/1.73 m2. ACE-inhibitor use did not change point estimates when included in the model and was removed.

GFR < 60 ml/min/1.73 m^2 ^by the 4-variable MDRD equation was considered renal dysfunction. Coronary heart disease was defined as history of confirmed myocardial infarction, or self-reported coronary revascularization or angina.

For the outcome of CHD, the *AGT1R *1166 C-allele in dominant model was associated with a significantly higher odds ratio in men while the *AGT *SNP M235T was associated with a significantly higher odds ratio in women (Table 2). Recessive inheritance modeling was borderline significant only for the *AGT *M235T (adjusted OR 1.52 [0.98 to 2.35]) for the presence of CHD in women. No recessive models were significant for men, and all additive models were null for both groups. Multivariable models that did not include prevalent hypertension yielded the same results as the fully adjusted models. Excluding eGFR < 60 ml/min/1.73 m^2 ^as a covariate in the models for CHD also did not influence the associations seen.

The *AGT *235 T-allele had a borderline association with history of CHD (OR 1.24 [0.96, 1.61] in the pooled data (Figure 1). The fully adjusted odds ratios for the combined data were not notably different from the age-adjusted only odds ratios (all differences resulted in < 10% change in OR). Results for CHD outcome did vary by sex; we observed significant interaction term p-values for the *AGT1R *1166 C-allele (p = 0.008) and *AGT *235 T-allele (p = 0.03) in relation to CHD (Table 2). In the combined data set, no significant associations were observed in recessive or additive models for CHD.

Because different allelic frequencies may be seen in different ethnic populations, we also analyzed these data with the few African-Americans excluded. Exclusion of these participants did not meaningfully change any of the results in the analyses stratified by sex or in the combined cohort. For example, the odds ratio for *AGT1R *A1166 C-allele was 1.70 [1.04, 2.78] for eGFR < 60 ml/min/1.73 m^2 ^and 1.56 [1.09, 2.21]for CHD when African-Americans were excluded from the HPFS analyses. Similarly in the combined cohort, the odds ratio for *AGT1R *A1166 C-allele and eGFR < 60 ml/min/1.73 m^2 ^was 1.42 [1.02 to 1.98].

## Discussion

The *AGT1R *1166 C-allele was directly and significantly associated with renal dysfunction in the combined dataset. This SNP was also associated with higher odds ratio for prevalent CHD in men with type 2 diabetes whereas the AGT 235 T-allele was significantly associated with increased odds ratio for CHD in women with type 2 diabetes. In contrast to the majority of previous publications of *AGT1R *and *AGT *polymorphisms, this study only included adults with type 2 diabetes, which is a population especially at high risk for kidney dysfunction and CHD.

We also found that sex might modify the association between SNPs and presence of CHD, and recommend that future investigations consider stratifying analyses by sex. A biological basis for these observed sex differences may be indirectly supported by previous studies of the RAS. For example, a population study of several hundred Bavarians revealed higher renin and prorenin levels in men compared with women; the authors also noted the lowest renin and prorenin levels in women taking hormone replacement therapy and hypothesized that sex hormones may regulate expression of the renin gene [24]. Healthy men also exhibit greater decreases in plasma renin and angiotensin-II levels than healthy women when given angiotensin-receptor blocker medications [25]. Another investigation of 93 healthy young men and women also reported higher levels of angiotensin-(1–7) peptides in men compared to women, but did not detect significant differences in levels of A-I, A-II, or angiotensin-(1–7) between the sexes when stratified by *AGT1R *1166 C-allele or AGT M235T genotypes [26]; however, the study had low power because many subgroups analyzed had less than 10 people.

We examined dominant, recessive, and additive genetic models for each of these four SNPs and eGFR< 60 ml/min/1.73 m^2 ^and CHD and did not detect significant associations with recessive models or additive models. Previous analyses of the *AGT *M235T SNP and CHD have reported an association with the homozygous T/T variant [10,13,14], but these relatively small studies may not have had enough power to effectively identify recessive genetic effects.

Our findings that the *AGT1R *1166 C-allele was associated with renal dysfunction in men and women with type 2 diabetes is consistent with a smaller French study of 235 type 2 diabetics where the authors reported that the CC genotype was significantly associated with nephropathy (defined as presence of microalbuminuria) especially in men[27]. Although previous case-control studies demonstrated an increased frequency of the *AGT1R *1166 C-allele in ESRD patients with [28] and without type 2 diabetes [29] compared to healthy controls, our study appears to be one of the few to examine an earlier stage of moderate kidney dysfunction. In contrast, other studies have not found association between A1166 C-allele and nephropathy in type 2 diabetes [30,31].

Consistent with our findings, however, a study of 132 Japanese adults with type 2 diabetes [32], a Hong Kong study of 168 type 2 diabetics [33], a study from Germany of 301 type 2 diabetics [34], and a meta-analysis of several thousand people [35] reported no association between nephropathy and the *AGT *M235T polymorphism. In contrast, a recent investigation of 421 Asian Indian type 2 diabetics did find an association between the *AGT *235 T-allele and renal insufficiency (defined as plasma creatinine ≥ 3 mg/dl and/or urinary albumin excretion rate > 200 mcg/min) (OR 2.68, 95%CI 2.01 to 3.57) [36].

Direct comparisons of conflicting reports in the literature, however, may be difficult and discrepancies could be due to many factors such different prevalence in genetic polymorphisms in different ethnic populations as well as varying definitions of nephropathy (impaired eGFR or CrCl in our study vs. microalbuminuria in other studies) and varying sample sizes. For example, the frequencies of the *AGT *235 T-allele and the *AGT *(-6) G-allele appear to be much higher in Japanese compared with Caucasian populations [12].

Interestingly, the associations observed between these candidate RAS SNPs and renal dysfunction were not meaningfully changed after multivariable adjustment. In particular, excluding hypertension as a covariate from the model did not change results and support the concept that association between RAS SNPs (and presumed upregulation of the RAS) and renal and CHD outcomes may be mediated by other influences in besides hypertension, which likely include cellular hyperplasia, stimulation of hormonal pathways and direct pro-fibrotic effects on the kidneys and vasculature [11]. Other investigators have also reported that hypertension may not mediate associations between these SNPs and CHD [13] or progressive CKD [37]. Also notable is the lack of influence of including eGFR < 60 ml/min/1.73 m^2 ^as a covariate in models where CHD was the dependent variable, which may suggest that renal dysfunction has a weaker association with CHD in type 2 diabetes (who have often have multiple overlapping vascular risk factors) than in non-diabetic populations.

Some important limitations of this investigation need to be acknowledged. Data on albuminuria, another measure of kidney dysfunction in addition to eGFR or CrCl were not available in the men and the majority of the women. Kidney function was not directly measured but estimated by a single measurement of plasma creatinine in each participant. The participants were mostly Caucasians and the findings may not be generalizable to other ethnic groups. There are no measurements of plasma angiotensiongen levels or other markers of RAS activation available to correlate directly with the genetic polymorphisms investigated here. The relatively small sample sizes and few numbers of participants meeting criteria for kidney dysfunction has resulted in wide confidence intervals for many of the estimates for eGFR < 60 ml/min/1.73 m^2^. Also, as the DNA was collected several years after the initiation of the cohort study, there is a possibility that participants who died prior to the blood collection may have had the candidate SNPs; this situation would be expected to bias results towards the null, however, whereas we did observe a number of significant associations.

Strengths of the current study include the examination of genetic polymorphisms in the RAS that have received less attention than the ACE-I/D genotype, which provide novel information concerning genetics and kidney and cardiac disease in adults with type 2 diabetes. Furthermore, by examining the SNPs separately in a cohort of men and one of women as well as in a combined dataset, these analyses revealed that sex may modify the association between RAS gene polymorphisms and coronary heart disease in type 2 diabetes.

## Conclusion

In conclusion, among type 2 diabetics, the *AGT1R *1166 C-allele is directly associated with kidney dysfunction in men and women but with CHD only in men. The *AGT *235 T-allele is associated with CHD in women. Previous evidence suggests that these polymorphisms are associated with up-regulation of the RAS, and therefore, these data further support the central role of RAS activation in CKD and CHD.

## Competing interests

The authors declare that they have no competing interests.

## Authors' contributions

JL conceived of the study, obtained funding for the genotyping, performed statistical analyses, and drafted the manuscript. FBH established the HPFS and NHS diabetes subcohorts and obtained funding for DNA extraction. LQ participated in statistical analyses and edited the manuscript. GCC participated in the design of the study and edited the manuscript. All authors read and approved the final manuscript.

## Pre-publication history

The pre-publication history for this paper can be accessed here:

http://www.biomedcentral.com/1471-2369/10/9/prepub
